# Protocol Improvement for RNA Extraction From Compromised Frozen Specimens Generated in Austere Conditions: A Path Forward to Transcriptomics-Pathology Systems Integration

**DOI:** 10.3389/fmolb.2020.00142

**Published:** 2020-07-22

**Authors:** Nabarun Chakraborty, Connie W. Schmitt, Cary L. Honnold, Candace Moyler, Stephen Butler, Hisham Nachabe, Aarti Gautam, Rasha Hammamieh

**Affiliations:** ^1^Geneva Foundation, Walter Reed Army Institute of Research, Silver Spring, MD, United States; ^2^Medical Readiness Systems Biology, Walter Reed Army Institute of Research, Silver Spring, MD, United States; ^3^Comparative Pathology, US Army Medical Research Institute of Chemical Defense, Gunpowder, MD, United States; ^4^ORISE, Walter Reed Army Institute of Research, Silver Spring, MD, United States

**Keywords:** assay optimization, histopatholgy, transcriptomics, product development, systems integration

## Abstract

At the heart of the phenome-to-genome approach is high throughput assays, which are liable to produce false results. This risk can be mitigated by minimizing the sample bias, specifically, recycling the same tissue specimen for both phenotypic and genotypic investigations. Therefore, our aim is to suggest a methodology of obtaining robust results from frozen specimens of compromised quality, particularly if the sample is produced in conditions with limited resources. For example, generating samples at the International Space Station (ISS) is challenging because the time and laboratory footprint allotted to a project can get expensive. In an effort to be economical with available resources, snap-frozen euthanized mice are the straightforward solution; however, this method increases the risk of temperature abuse during the thawing process at the beginning of the tissue collection. We found that prolonged immersion of snap frozen mouse carcass in 10% neutral buffered formalin at 4°C yielded minimal microscopic signs of ice crystallization and delivered tissues with histomorphology that is optimal for hematoxylin and eosin (H&E) staining and fixation on glass slides. We further optimized a method to sequester the tissue specimen from the H&E slides using an incubator shaker. Using this method, we were able to recover an optimal amount of RNA that could be used for downstream transcriptomics assays. Overall, we demonstrated a protocol that enables us to maximize scientific values from tissues collected in austere condition. Furthermore, our protocol can suggest an improvement in the spatial resolution of transcriptomic assays.

## Introduction

The value added from big data is in generating information and, ultimately, knowledge by integrating a large amount of reads derived from multiple disparate assay types (Marx, 2013a; Andreu-Perez et al., 2015; Gligorijević et al., 2016; Huang et al., 2017). This is due to the distinct data types that may require different and incompatible sample processing methods. It can be difficult to obtain all the desired data from a single specimen (Bean et al., 2012; Yan et al., 2015). Moreover, analyzing similar but not identical specimens can lead to spurious results in this type of multi-modal data acquisition (Bean et al., 2012; Gligorijević et al., 2016). For example, dissecting a partially damaged whole tissue into multiple fragments for gene expression and histopathological assessment may confound the analysis, if a pathological lesion does not extend uniformly across all the fragments, or if there are subtle spatial variations in the tissue, as has been shown for tumors (Yan et al., 2015). This drawback attributed to the intrinsic heterogeneity of tissues that can potentially be mitigated by integrating the omics data with other “conventional” readouts (Ellinger-Ziegelbauer et al., 2011), such as histopathology analysis. An omics-pathology integration approach could be the most effective for phenome-to-genome interpretation if omics assays are conducted using the particular tissue specimen, where injury signatures are informed by histopathology image analysis (Pathak and Dave, 2014; Yu et al., 2017). This approach would be an ideal phenome-to-genome approach, where omics readouts could be directly informed by the visualized phenotypes (Yee, 2009; Gligorijević et al., 2016). In such an instance, there is an obvious and clear advantage in recycling the same tissue specimens for both gene expression and histopathology image analyses. Multiple assay data generated from the same tissue specimen will improve spatial resolution of tissue's molecular landscape, enhance data integrative structure, and reduce biases in generating multi-dimensional information, which is the goal of making big data free from false results (Marx, 2013b; Andreu-Perez et al., 2015; Gligorijević et al., 2016; Huang et al., 2017).

There is a paramount need for a platform that is capable of deriving maximum information from a minimum amount of samples in space biology, as space-flown samples are scarcer. Moreover, samples harvested from space-flown specimens provide uniquely valuable insights and should be exploited by multi-dimensional genome-to-phenome analysis. Handling animals in microgravity is immensely challenging, which is further aggravated when the animals need to be operated on using an aseptic technique (Choi et al., 2015; Globus et al., 2015, 2016). Based on our own experience (Dadwal et al., 2019), the estimated time for handling each mouse on the International Space Station (ISS) was five to six times longer than that required on the ground. In addition, investigators need to make every effort to minimize the physical footprint taken on a spaceflight by a given specimen and its handling equipment. To satisfy all of these constraints, freezing the animal carcass in space without any further dissection was considered the most prudent compromise. These snap-frozen carcasses had to undergo on-ground freeze-thaw cycles before tissue collection, which is typically expected to compromise overall sample quality and make the histopathologic analysis challenging (Lyons et al., 1979; Pikal-Cleland et al., 2000). Increased level of interest is evident in recent years to integrate the omics data with histopathology readouts (Brenna et al., 2013; Murata et al., 2015); however these studies were typically at a risk of specimen or animal bias, since different cohorts of animals were used for omics and pathology assays, respectively. Acknowledging this knowledge gap, several techniques have been developing to improve spatial, cellular, and sub-cellular resolution of transcriptomics readouts (Grün and van Oudenaarden, 2015; Mignardi et al., 2016; Dewez et al., 2019).

Working on a similar objective, present manuscript demonstrates a protocol optimized for handling frozen samples. Notably, there are several tissue preservation protocols and agents available for long term storage of biomolecules, such as RNA, DNA, and proteins (Florell et al., 2001; Vincek et al., 2003); however, we plan to snap freeze the tissue without using any preservatives to best simulate those austere situations (Dadwal et al., 2019), where various preservatives are often unacceptable owing to their levels of toxicity or viscosity or difficulty in general handling. In this context, we proposed three strategies to thaw pre-frozen specimens. We present all three strategies, as well as our recommendations after considering the advantages and disadvantages associated with each individual method. Finally, we utilized recently available commercial kits to extract RNA from fixed, processed tissue slices mounted on standard glass histology slides. Altogether, this work is a demonstration of an optimized protocol for working with frozen samples of limited quantity collected in austere conditions.

## Materials and Methods

All animal experiments were approved by the Institutional Animal Care and Use Committee (IACUC) at the US Army Center for Environmental Health Research (USACEHR), Ft. Detrick, MD, and were performed in a facility accredited by the Association for the Assessment and Accreditation of Laboratory Animal Care International (AAALAC).

C57BL/6J mice, aged 6 weeks, were purchased from Jackson Labs, ME. Three animals were euthanized with an injection of ketamine-xylazine (150/45 mg/kg of mouse bodyweight). Immediately after euthanasia, carcasses were transferred to a −80°C freezer to simulate freezing of tissues during a space flight. All the mice carcasses were stored in the same box. After 7 days, the carcasses were removed from the freezer, and one of the three thawing strategies was applied.

### Thawing Strategy Options

**Figure 1:**
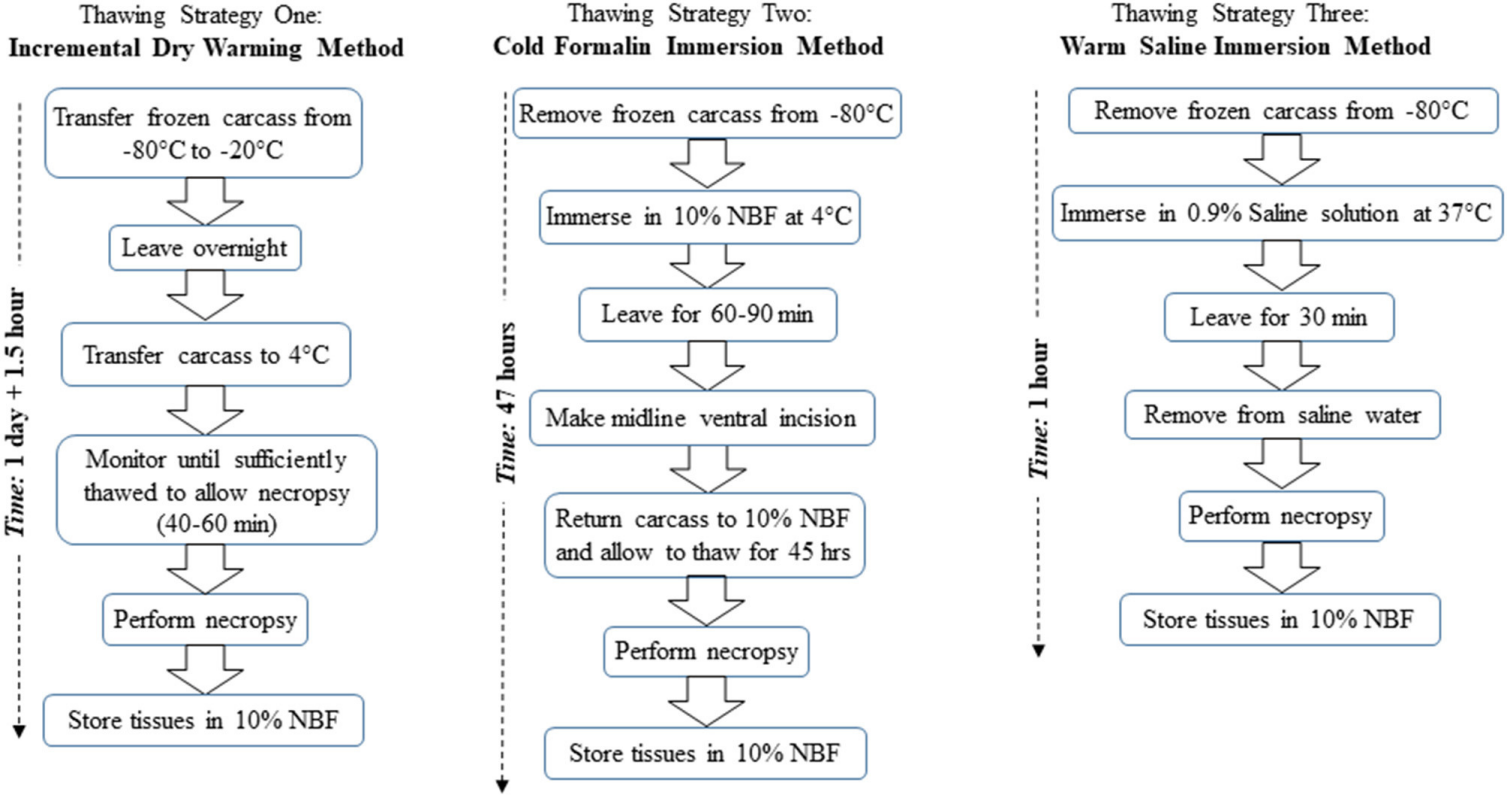
Flow diagram overview of the three thawing strategies, namely, strategy one, incremental dry warming method; strategy two, cold formalin immersion method; and strategy three, warm saline immersion method. NBF refers to neutral buffered formalin.

Figure 1 provides a flow diagram overview for each of the three thawing strategies described below.

#### Thawing Strategy One: Incremental Dry Warming Method

Following a 7-days holding period at −80°C, three mouse carcasses were transferred to a −20°C freezer and held overnight to incrementally thaw the tissues. Direct transfer from one freezer to the other was crucial to prevent excessive thawing of the carcasses at room temperature. The following morning, the carcasses were transferred from the −20°C freezer to a 4°C ice bucket. They were then closely monitored for 40–60 min until sufficiently thawed to perform necropsy. For necropsy, the carcass should ideally have a firm consistency to allow proper handling of internal organs, but not have thawed to the extent that the tissues are too soft to maintain integrity during handling. Following thawing, a comprehensive necropsy was performed.

#### Thawing Strategy Two: Cold Formalin Immersion Method

Following a 7-days holding period at −80°C, three mouse carcasses were placed in 10% neutral buffered formalin (NBF) solution at 4°C. After 60–90 min in 10% NBF, the carcasses were partially thawed. The carcasses were briefly removed from the 10% NBF to a sterilized plate, and a ventral midline incision was made through the skin and abdominal wall to facilitate penetration of the fixative into the abdominal cavity and internal organs. After making the midline incision, the carcass was returned to the 4°C 10% NBF solution for an additional 45 h. After the 45 h elapsed, the carcass was removed from the fixative, and a comprehensive necropsy was performed.

#### Thawing Strategy Three: Warm Saline Immersion Method

A 500 mL container was filled with 300 mL of 0.9% saline solution (Sodium Chloride, Fisher Scientific, Pittsburgh, PA, stock solution) at 37°C. Following a 7-days holding period in −80°C, three mouse carcasses were submerged in the warm (i.e., 37°C) saline solution for 30 min to facilitate thawing. Upon removal from saline solution, a comprehensive necropsy was performed.

### Necropsy

Post-thawing, the following tissues were collected: lacrimal gland, eye, brain, tongue, salivary gland, mandibular lymph node, nasal cavity, trachea, esophagus, heart, aorta, thymus, axillary lymph node, lungs, liver, gallbladder, pancreas, spleen, adrenal gland, kidney, mesenteric lymph node, inguinal lymph node, urinary bladder, stomach, duodenum, jejunum, ileum, cecum, colon, rectum, skeletal muscle, sternum, peripheral nerve, cervical, thoracic, and lumbar spinal cord. These tissues were preserved in 10% NBF (Fisher Scientific, Pittsburgh, PA). A subset of these tissues were utilized for subsequent downstream analysis (Table 1). The necropsy procedure used for tissue collection was consistent across all three thawing strategies.

**Table 1:** Quality and concentration of RNA extracted from different H&E fixed tissue slices collected from 3 mice.

**Tissue**	**Thawing strategy**	**Conc. (ng/μL)**	**260/280**	**260/230**	**RIN**	**DV_**200**_**
Midbrain	(1) Incremental drying	5.90 ± 0.90	2.31 ± 0.81	0.41 ± 0.08	4.0 ± 1.08	41.93 ± 2.64
	(2) Cold formalin immersion	20.70 ± 2.28	1.79 ± 0.02	0.10 ± 0.002	3.5 ± 1.75	14.17 ± 2.57
	(3) Warm saline immersion	67.26 ± 8.01	1.48 ± 0.90	0.68 ± 0.005	3.1 ± 1.81	22.64 ± 7.25
Sternum	(1) Incremental drying	32.70 ± 2.81	1.60 ± 0.006	0.68 ± 0.08	4.4 ± 0.95	20.35 ± 2.65
	(2) Cold formalin immersion	13.00 ± 3.20	1.42 ± 0.03	0.33 ± 0.01	4.2 ± 0.86	14.76 ± 7.06
	(3) Warm saline immersion	16.06 ± 4.66	1.48 ± 0.22	0.43 ± 0.89	4.4 ± 1.37	24.0 ± 1.08
Spinal Cord, cervical, thoracic and lumber segments	(1) Incremental drying	79.56 ± 8.87	1.52 ± 0.05	0.66 ± 0.007	3.4 ± 0.92	19.07 ± 8.47
	(2) Cold formalin immersion	30.54 ± 9.01	1.57 ± 0.42	0.38 ± 0.09	4.4 ± 1.05	27.60 ± 3.73
	(3) Warm saline immersion	29.94 ± 8.06	1.69 ± 0.47	0.39 ± 0.40	3.3 ± 0.46	22.25 ± 1.50

*All tissues were extracted from pre-frozen carcasses following one of the three thawing strategies, specifically, #1 Incremental Drying, #2 Cold Formalin Immersion, and #3 Warm Saline Immersion. The average values are presented with standard deviations in the format mean ± standard deviation*.

### Routine Tissue Processing

Following necropsy and fixation, tissues received a gross inspection, placed in tissue cassettes (Fisher Scientific, Pittsburgh, PA), and routinely processed. In detail, tissues with 5 micron thickness were paraffin-embedded, sectioned, mounted, stained with hematoxylin and eosin (H&E), followed by the application of a coverslip, and then allowed to dry at room temperature prior to microscopic evaluation (Dey, 2018). All tissues were evaluated microscopically by a board-certified veterinary pathologist using representative H&E stained tissue sections.

### Tissue Collection From Glass Histopathology Slides

The tissues were subjected to two techniques of RNA extraction from the histopathology slides. These techniques are described in the sections that follow.

#### Tissue Sequestration Strategy One: Xylene at 21°C With Liquid N_2_

Tissue was isolated from the processed slides by incubating them in xylene (Sigma-Aldrich, Saint Louis MO) and using a Wheaton Slide Preparation System Glass Staining Dish in a biological safety cabinet at room temperature. Different lengths of incubations of slides in xylene at 21°C were tried (starting at 20 min, then 10 min more and finally an overnight incubation) separated by incubations at room temperature for 30 min without xylene. This was combined with removal strategies using (i) a razor or scalpel blade and (ii) encasing slides in Styrofoam and repeatedly dipping them in liquid nitrogen to coax the tissue specimen off via cold snapping.

#### Tissue Sequestration Strategy Two: Xylene at 37°C

All histology slides were deparaffinized using xylene (Sigma-Aldrich, Saint Louis MO). The slides were submerged in xylene in a Wheaton Slide Preparation System Glass Staining Dish and covered. The samples in the staining dish were placed into a biological safety cabinet for subsequent steps. The slides were shaken in a 37°C incubator shaker (Boekel Industries Inc.) for 20 min. To remove the samples from the glass slides, we used a single-edged industrial razor blade made from surgical carbon (International West Chester, PA). After the incubation period, we used the sterile single-edged razor to remove the sample from each slide.

### RNA Isolation and Evaluation of RNA Quality and Quantity

RNA was isolated using the AllPrep DNA/RNA FFPE Kit (Qiagen, Carlsbad CA) as per the vendor's recommendations. The RNA concentration was measured using a Thermo Scientific NanoDrop 8000 spectrophotometer (Waltham, MA), and RNA quality was assessed using the Agilent Tapestation 2200 (Santa Clara, CA). Using a Qubit fluorometer (Invitrogen) a high sensitivity assay was conducted to further validate the concentration. After evaluation, the RNA was stored at −80°C.

## Results and Discussion

Freezing artifacts is characterized by the disruption of cellular membranes or tissues due to the formation of ice crystals within intra- or extracellular spaces. One of the most common causes of freezing artifacts is temperature abuse, which occurs when fixed wet tissue is allowed to freeze, thaw, and refreeze, fluctuating repeatedly above and below the freezing point of the fixative (Xiong, 1997). For example this may result when tissue is held in a cooler that fails to maintain a constant temperature above the freezing point of the fixative Adams, 2007. Moreover, distortion or damage to cellular constituents caused by the ice crystal formation and moisture migration can confound the omics evaluation of tissues. For the present protocol, mice were flash frozen; thus, the initial risk of temperature abuse was potentially averted. Minimization of temperature abuse during the thawing process remained our primary challenge.

**Figure 2:**
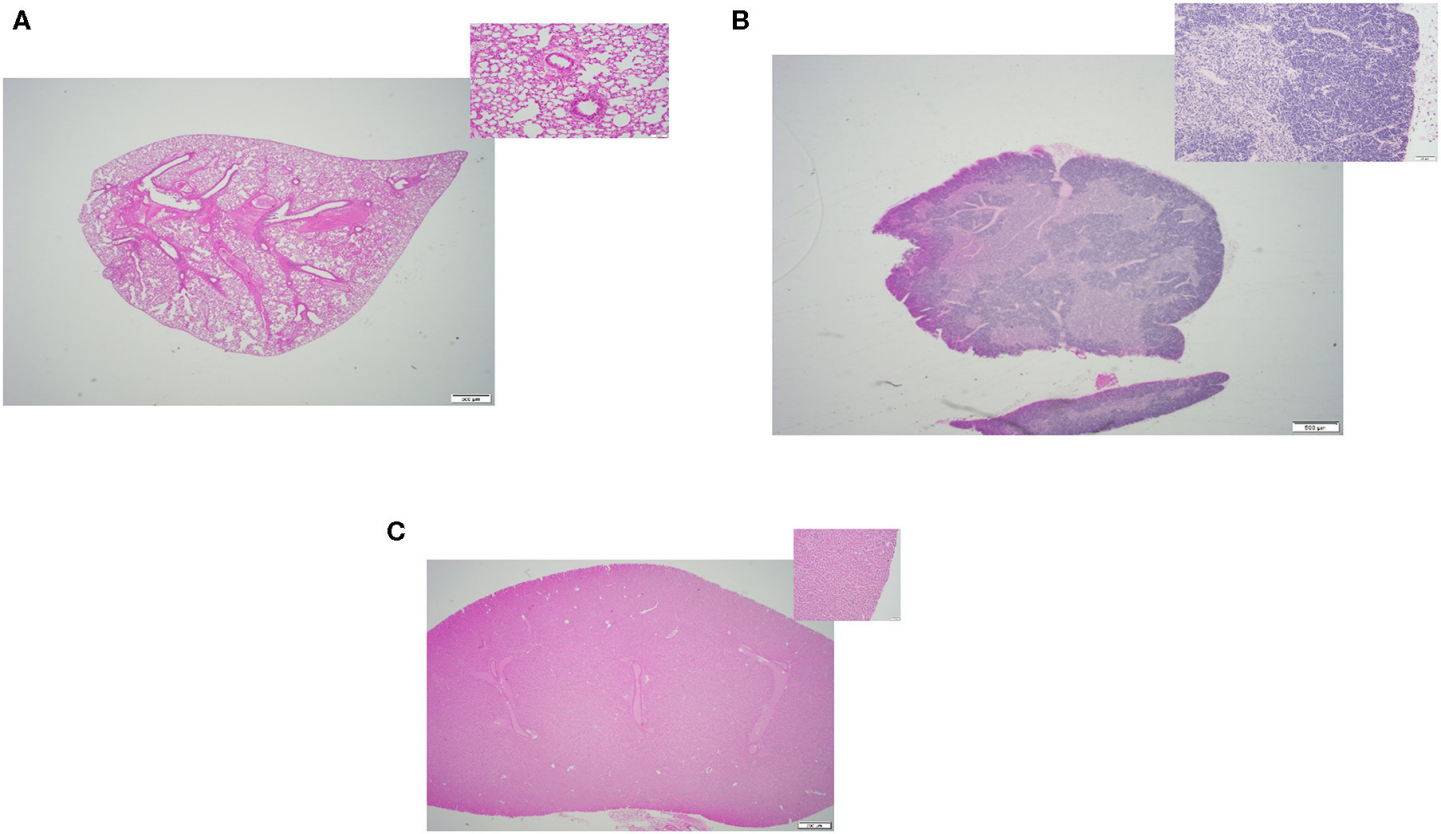
Representative H&E slides prepared from mouse tissue samples collected following thawing strategy one, incremental dry warming method. **(A)** Lung tissue (20x) and its inset: 200x magnification of the same tissue. **(B)** Thymus tissue (20x) and inset: 200x magnification of the same tissue; **(C)** Kidney tissue (20x) and its inset: 200x magnification of the same tissue.

**Figure 3:**
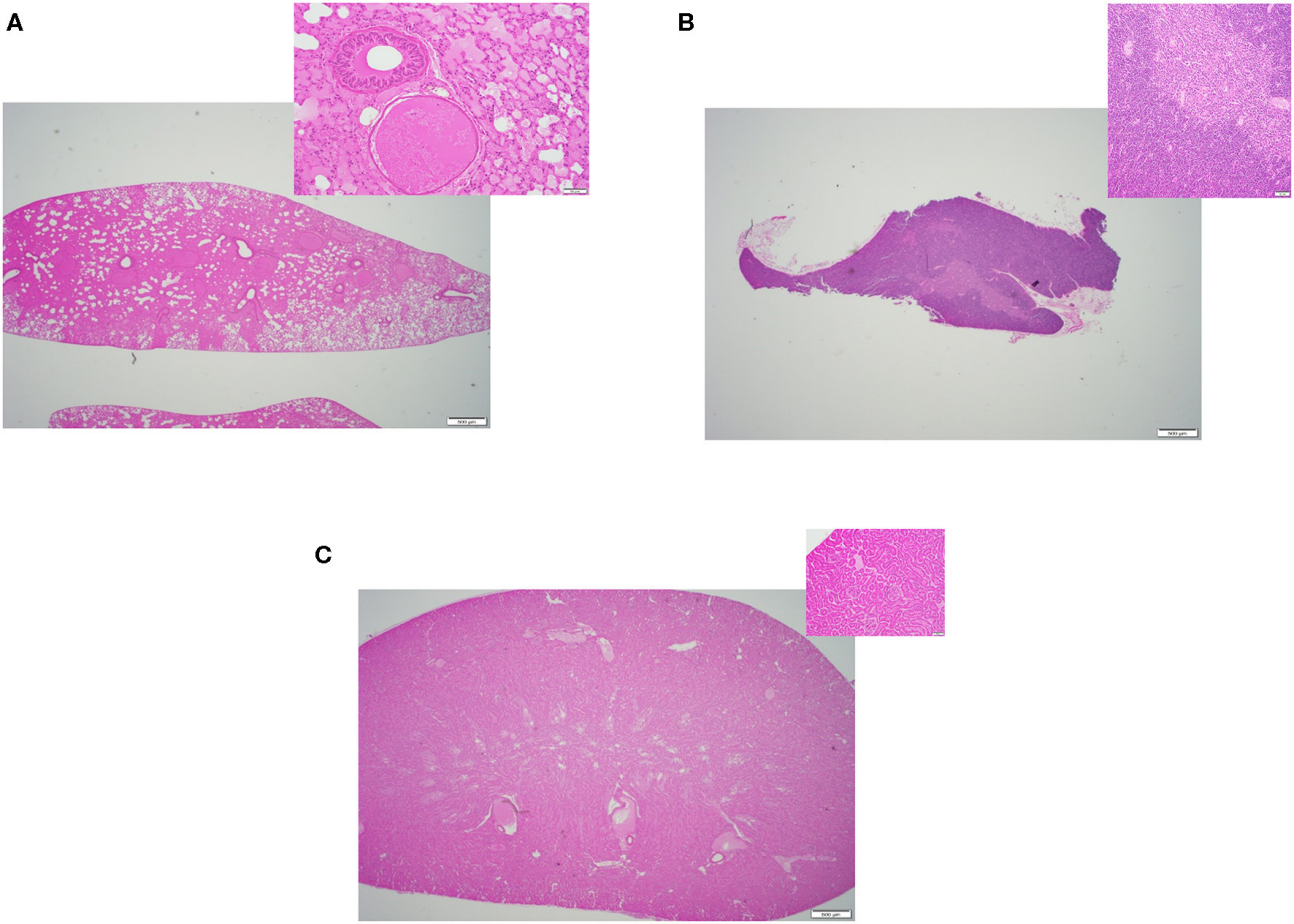
Representative H&E slides prepared from mouse tissue samples collected following thawing strategy two, cold formalin immersion method. **(A)** Lung tissue (20x) and its inset: 200x magnification of the same tissue. **(B)** Thymus tissue (20x) and inset: 200x magnification of the same tissue; **(C)** Kidney tissue (20x) and its inset: 200x magnification of the same tissue.

**Figure 4:**
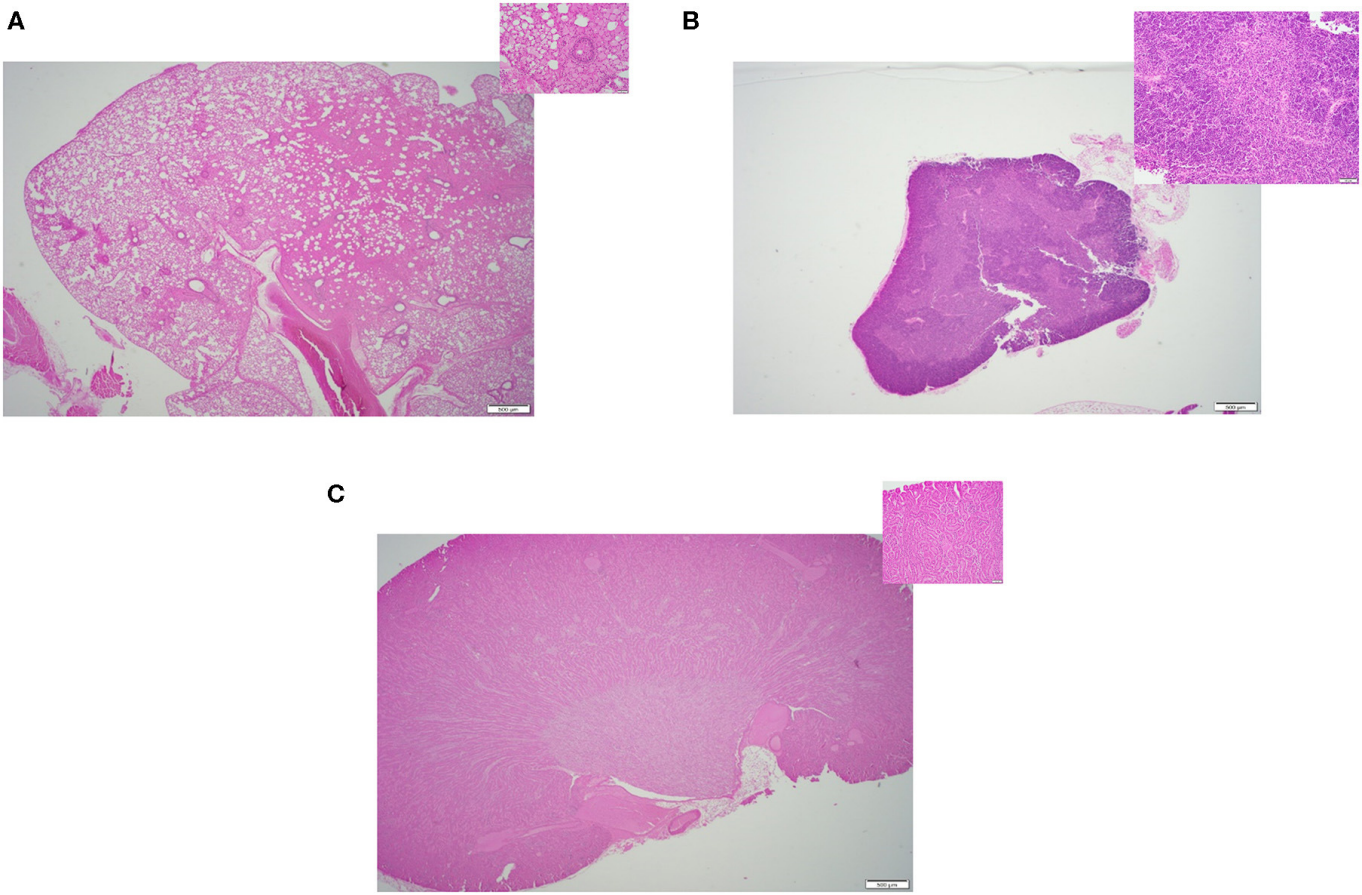
Representative H&E slides prepared from mouse tissue samples collected following thawing strategy three, warm saline immersion method. **(A)** Lung tissue (20x) and its inset: 200x magnification of the same tissue. **(B)** Thymus tissue (20x) and inset: 200x magnification of the same tissue; **(C)** Kidney tissue (20x) and its inset: 200x magnification of the same tissue.

In this study, we compared the efficacy of three different methods of thawing frozen mouse carcasses. Criteria of success included: (i) Tissue quality- the overall quality of the tissues, any sign of damages or lesions were initially inspected by a board-certified veterinary pathologist through a microscope. Figures 2–**4** displays the representative pictures of the tissues with two different magnifications, respectively. Furthermore, we evaluated the qualities of RNA extracted from these individual tissues. RNA integrity numbers (RIN) and DV_200_ (i.e., the percentage measure of RNA fragments >200 nucleotides) were used as the benchmarks to determine RNA quality and in retrospect, the tissue quality. (ii) Time expended with particular focus on the hands-on time during the thawing process, and (iii) implementation ease, which was primarily dependent on subjective evaluation by our technical staff.

### Tissue Quality

All three thawing strategies yielded similar results in terms of final tissue quality (see Figures 2A–C, 3A–C, 4A–C). No damages or lesions were found. Table 1 describes the qualities and quantities of RNA samples extracted from the tissues, which was discussed in detail in the subsequent section evaluation of the sequestration processes.

### Time Expended

In terms of time expended for each strategy, strategy three, Warm Saline Immersion, had the quickest overall turnaround time, requiring only 30 min per carcass for the thawing process with a minimal handling time of <2 min prior to the start of the necropsy procedure. Strategies one and two, required significantly more overall time for the thawing process, 25.5 and 47 h, respectively, but only about 20 min of actual hands-on time per carcass during the process. Once thawing was complete, the total necropsy time (i.e., ~30 min), was similar for all three strategies.

### Implementation Ease

In terms of implementation ease, strategy one, Incremental Dry Warming, resulted in a dry, thawed, unfixed carcass which was relatively fragile when compared to a routine fresh carcass. Tissues were susceptible to artifactual damage, such as tearing of skin and tubular organs as well as whole-organ breakage, during examination and handling using normal necropsy instruments. Extra care during tissue handling was needed to avoid tissue damage, and to retain whole-organ tissue architecture for handling, collection, and processing prior to fixation.

In contrast, strategy two, Cold Formalin Immersion, resulted in a wet, thawed, fixed carcass that retained solid tissue architecture and was resistant to introduction of damage during examination and handling using normal necropsy instruments. Starting the necropsy with a thawed, fixed carcass allowed for relatively uncomplicated tissue handling and collection prior to further processing.

Strategy three, Warm Saline Immersion, resulted in a wet, thawed, unfixed carcass which most closely resembled that of a routine fresh carcass compared to the other two strategies. However, wet, warm, unfixed tissues became soft, which allowed for increased water content, making them susceptible to damage such as shearing or tearing of solid organs, and ripping of skin and tubular organs during examination and handling using normal necropsy instruments. Extra care during tissue handling and collection was required to avoid tissue damage and loss of tissue architecture prior to fixation.

Subsequently, all the tissues of interest were fixed in 10% neutral buffered formalin, grossed, placed in cassettes, and then routinely processed, sectioned to a thickness of 5 microns, and stained using hematoxylin and eosin (Ellinger-Ziegelbauer et al., 2011). Next, we selected those tissues, such as bone and muscle, which are typically challenging tissues to extract RNA from. The primary challenge in this tissue sequestration protocol was optimizing an effective method to recover tissue sections from a prepared and stained glass histopathology slide. Two methods for removing tissue from the slide using xylene were tested. For the first method, the slides were incubated with xylene at 21°C over multiple incubation periods; despite these incubations as well as cold snapping, we were unable to separate the tissue from the slide. Hence, this tissue sequestration strategy was abandoned. The second method was successful; it involved incubating the slides with xylene at a higher temperature, specifically at 37°C.

RNA was extracted from the tissue specimens sequestered by the second method, and Table 1 shows the qualities and quantities of these RNA samples. The RNA qualities and quantities were comparable across three thawing strategies. The RIN values were consistently around 4 and DV_200_ values were lower than 30; and these suboptimal scores could be attributed to the compound effects of several factors, including the compromised tissue preservation method and the effect on RNA quality due to NBF (cross-linking and direct reaction with nucleotides; Cox et al., 2006). By certain account, the DV_200_ score is the fittest metric to decide upon the appropriateness of the samples to run for gene expression analysis (Matsubara et al., 2020); while the prevalent thought is to consider both RIN and DV_200_ to finalize this decision. Using RNA samples with RIN score >2.0, Ravo et al. claimed a complete success in microarray-based cDNA-mediated annealing, selection, extension and ligation (DASL) assay; furthermore, these authors achieved similar success in DASL assays using samples with RIN <2.0 but the majority of RNA size was >200 nt (Ravo et al., 2008). This concept was adapted to achieve an uniform gene sequencing coverage using RNA samples with RIN scores between 2.0 and 4.0 (Cieslik et al., 2015). Other study reported nearly 90% mapping rate to human genome using RNA sample with RIN and DV_200_ scores equaled to 2.3 and 29, respectively (Lin et al., 2019). In this context, a number of commercial kits have been evaluated to score their merits in sequencing low quality RNA samples (Adiconis et al., 2013; Kresse et al., 2018; Masters et al., 2018; Lin et al., 2019) and these feedbacks could be used as the benchmark for effectively analyzing the compromised RNA specimens. Selected from this pool of kits, we have successfully used NuGEN kit (NuGEN Technologies, San Carlos, CA), and Systems BioSciences kits (Systems BioSciences, Mountain View, CA) to enrich and amend severely compromised RNA specimens collected from the space-flown cells to produce cDNA microarray data (Chakraborty et al., 2014). Taking together, we believe that the RNA specimens collected from the present methods are eligible to be treated by these commercial kits for successful gene expression analysis.

Present approach has certain drawbacks. The design of this study was to simulate austere conditions in a space environment by snap freezing the carcasses without accessing any preservation agents and subjecting the samples to freeze-thaw cycles that would imitate sample processing during and after space flight. Actual space flight might display conditions not completely simulated by our model. Nevertheless, this model provides a reliable and reproducible analysis of plausible methods to best represent how the scientific data can be accurately captured. Our team has had previous exposures with space flight-related experiments (Dadwal et al., 2019) that supplied them with a breadth of virtues and pitfalls that could be realistically associated with these methods in an actual space flight experiment. The sample size of the present study (*n* = 3) was small but statistically viable. The histopathology slides were inspected by single board-certified pathologist, which is an accepted practice to assess quality of the histomorphology of a slide in both diagnostic and toxicological pathology settings. Furthermore, NBF is the sole source of fixative agents approved by our institute, hence present protocol precluded from testing the efficacy of any other alternatives (Kiernan, 2000).

In conclusion, this protocol could be a paradigm shift from the typical transcriptomics assay that use homogenized tissue specimens and thereby are often criticized to miss the localized signals of stress (Mignardi et al., 2016). Here we presented a scope to inspect the “localized signal” of partially damaged tissue through histopathology image analysis, and let the H&E information guide in selecting the most suitable tissue specimen for omics study. Our technology will be greatly beneficial when tissues are collected from austere or unique conditions such as spaceflight, War Theater and pandemic centers (such as the present COVID-19 situation), where we cannot afford to obtain multiple samples, but multiple assays are necessary to comprehend the holistic picture. Herein our method will inform how a single tissue can be sequentially used for multiple assays. Certainly, the motivation for this study was to develop a protocol best suited for processing frozen carcasses originating from the ISS; however, we can foresee that our final deliverables have potential applicability to additional research interests.

## Data Availability Statement

All datasets generated for this study are included in the article/supplementary material.

## Ethics Statement

The animal study was reviewed and approved by IACUC committee of USACEHR. Material has been reviewed by the Walter Reed Army Institute of Research. There is no objection to its presentation and/or publication. The opinions or assertions contained herein are the private views of the author, and are not to be construed as official, or as reflecting true views of the Department of the Army or the Department of Defense. Research was conducted under an approved animal use protocol in an AAALAC accredited facility in compliance with the Animal Welfare Act and other federal statutes and regulations relating to animals and experiments involving animals and adheres to principles stated in the Guide for the Care and Use of Laboratory Animals, NRC Publication, 2011 edition.

## Author Contributions

NC and RH conceived the idea. CS and CH conducted histopathology study. AG, SB, HN, and CM conducted molecular assays. AG, HN, and NC carried out analysis. NC and HN drafted the text. Everyone read, edited, and approved the report. All authors contributed to the article and approved the submitted version.

## Conflict of Interest

The authors declare that the research was conducted in the absence of any commercial or financial relationships that could be construed as a potential conflict of interest.
